# Solitary colonic polypoid ganglioneuroma

**DOI:** 10.1186/1746-1596-3-20

**Published:** 2008-04-29

**Authors:** Isabel Maria Mendez, Teresa Pereda, Francisco Javier Rodriguez, Rafael Funez, Andres Sanchez

**Affiliations:** 1 Service of Gastroenterology, Hospital Costa de Sol, Marbella, Malaga, Spain; 2 Service of Anatomic Pathology, Hospital Costa de Sol, Marbella, Malaga, Spain

## Abstract

This short report discusses a case of solitary colonic polypoid ganglioneuroma associated with melanosis coli in a woman with no systemic manifestations. To our knowledge this is the first ganglioneuroma reported in the literature in association with melanosis coli. The nature and significance of this event remains unclear, although this may be coincidental due to the laxative intake. Further investigation is necessary to clarify this point. The interest of this case lies moreover in the rarity of this entity and its endoscopic and histologic resemblance to sessile polyps frequent in the clinical practice.

## Findings

The hereditary syndromes of the gastrointestinal tract are classified as adenomatous or hamartomatous [1]. As a part of the hamartomatous polyposes, ganglioneuroma (GN) of the gastrointestinal tract are rare tumors composed of ganglion cells, nerve fibers, and supporting cells of the enteric nervous system [1,2]. There are few reports in the literature. We present a case of polypoid GN and melanosis coli. To our knowledge this is the first case reported in literature with this association.

**Figure 1:**
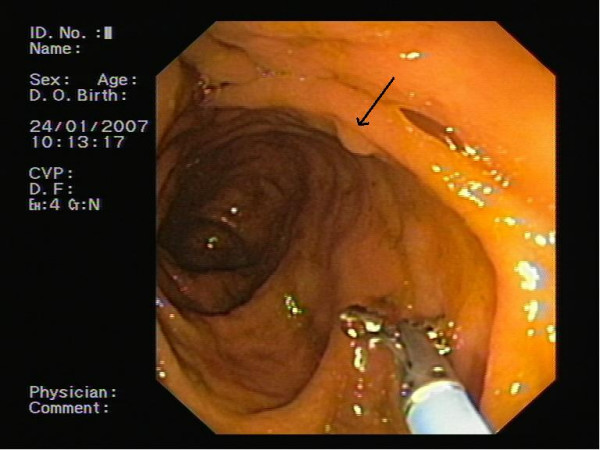
Endoscopic image of the polyp.

A 48-year old woman with arthrosis underwent colonoscopy because of a family history of colon cancer (father and cousin) and an episode of lower intestinal bleeding. The patient was an anthracene-type laxatives ("sacred rind") consumer due to usual constipation. No other symptoms were related. She and her family had no known history of multiple endrocrine neoplasia or neurofibromatosis. The laboratory tests were within the normal limits, including carcinoembryonic antigen (CEA). Physical examination revealed an anal fissure. No pigmented skin lesions were identified. The colonoscopy revealed a millimetric sessile polyp in the sigmoid colon that was removed with biopsy forceps (Figure 1).

**Figure 2:**
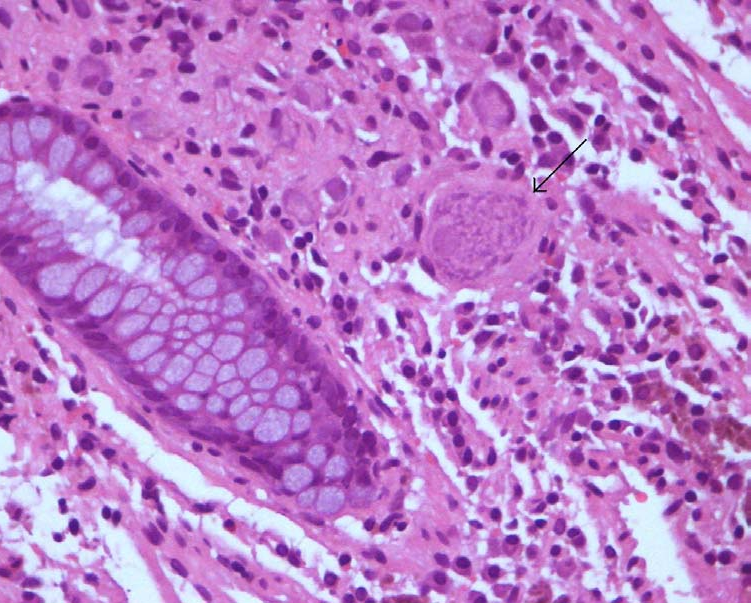
(H&E, ×40): High power microscopic view of the ganglion and stromal cells in the lamina propia. Melanosis coli associated.

**Figure 3:**
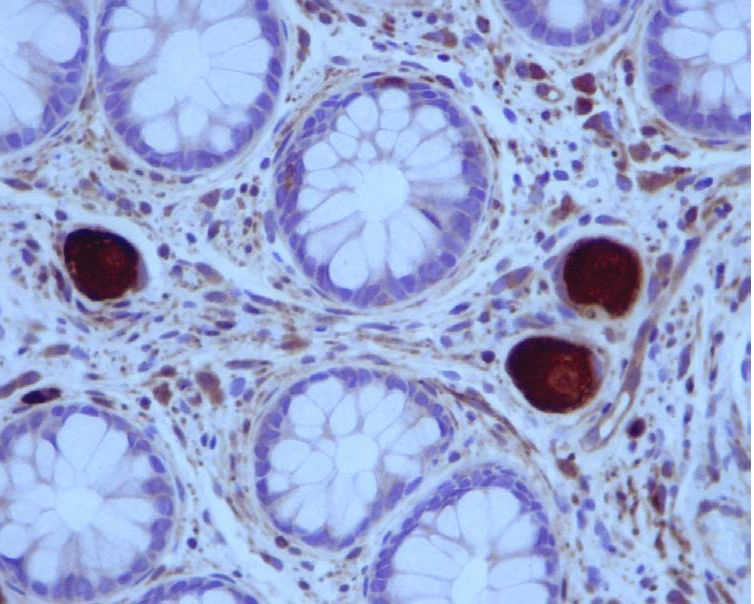
(NSE, ×40): Immunohistochemical staining of ganglion cells for NSE.

The biopsy specimen consisted of three fragments. At low magnification they looked like a hyperplastic polyp. Microscopic examination of the fragments of the polyp showed a collection of nerve ganglion and stromal cells in the lamina propia that elevated the overlying elongated glands in a nodular configuration. No significant disarray of the mucosal architecture was observed. An additional finding of diffuse melanosis coli was noticed (Figure 2). Inmunohistochemical studies were performed. The ganglion cells and the spindle stromal surrounding cells demonstrated inmunoreactivity to protein S-100, and neuron-specific enolase (NSE) marked the ganglion cells (Figure 3).

The diagnosis was solitary polypoid ganglioneuroma of the colon.

Shekita et al. categorized ganglioneuromas (GN) of the intestinal tract into three groups: polypoid GN, ganglioneuromatous polyposis and diffuse ganglioneuromatosis.

The GN of the gastrointestinal tract have been found in patients with familial adenomatosis coli, Cowden's disease, tuberous sclerosis, juvenile polyposis, von Recklinghausen's disease and multiple endocrine neoplasia type II [3]. The series by Shekita indicate that a solitary polypoid GN is not a sign for increased risk of Recklinghausen's disease or Multiple endocrine neoplasia (MEN) type II, but it has been reported in a few cases of Cowden's disease, tuberous sclerosis, polyposis coli and juvenile polyposis. In contrast, diffuse GN appears to be a strong factor for extensive neurogenic disease. In our case ganglioneuromas occurred without evidence of systemic disease.

Polypoid GN are small, sessile or pedunculated polyps with a histologic resemblance to hyperplastic polyps, juvenile polyps or adenomas [1,3]. In the intestinal tract, these lesions are solitary or few in number [1]. In the cases by Shekita et al, most of them were located in the large intestine [3]. Polypoid GN produces no characteristic symptoms and may be noted incidentally during endoscopy, surgery or autopsy. Abdominal pain, obstruction, constipation, ileus, appendicitis and weight loss have been listed as potential complications of the lesion, and all are related to their size and anatomical location [4].

The link between intestinal ganglioneuromatosis and malignancy is not well established, although some investigators have described the coexistence of intestinal ganglioneuromatosis and colorectal adenocarcinoma [5,6].

To our knowledge this is the first ganglioneuroma reported in the literature in association with melanosis coli. The nature and significance of this event remains unclear although this may be coincidental due to the laxative intake. Further investigation is necessary to clarify this point. The interest of this case lies moreover in the rarity of this entity and its endoscopic and histologic resemblance to sessile polyps frequent in the clinical practice.

## Abbreviations

GN: Ganglioneuroma; CEA: Carcinoembryonic antigen; NSE: Neuron-specific enolase; MEN: Multiple endocrine neoplasia.

## Competing interests

The authors declare that they have no competing interests.

## Authors' contributions

IMM and TP have contributed substantially to the conception and design of this work, giving final approval of the version to be published. IMM has attended the medical consultation of the patient and TP reported the pathologic diagnosis. Both edited the manuscript and made the required changes.

FJR, RF and AS have been involved in drafting the manuscript and revising it critically for important intellectual content.

All authors read and approved the final manuscript.
